# Sulforaphane-Loaded Ultradeformable Vesicles as A Potential Natural Nanomedicine for the Treatment of Skin Cancer Diseases

**DOI:** 10.3390/pharmaceutics12010006

**Published:** 2019-12-19

**Authors:** Maria Chiara Cristiano, Francesca Froiio, Roberta Spaccapelo, Antonia Mancuso, Steven P. Nisticò, Betty P. Udongo, Massimo Fresta, Donatella Paolino

**Affiliations:** 1 Department of Experimental and Clinical Medicine, University “Magna Græcia” of Catanzaro, Campus Universitario “S. Venuta”, viale Europa, 88100 Catanzaro, Italy; mchiara.cristiano@unicz.it (M.C.C.); f.froiio@unicz.it (F.F.); 2 Department of Experimental Medicine, University of Perugia, Piazzale Lucio Severi n. 1, 06132 Perugia, Italy; roberta.spaccapelo@unipg.it; 3 Department of Health Sciences, University “Magna Græcia” of Catanzaro, Campus Universitario “S. Venuta”, viale Europa, 88100 Catanzaro, Italy; antonia.mancuso@unicz.it (A.M.); nistico@unicz.it (S.P.N.); fresta@unicz.it (M.F.); 4 Pincer Training and Research Institute, Plot 1127, Lukuli Zone 5 00256, Uganda; bettyudongo@gmail.com

**Keywords:** sulphoraphane, ethosomes^®^, transfersomes^®^, human stratum corneum, percutaneous permeation, anticancer activity

## Abstract

Sulforaphane is a multi-action drug and its anticancer activity is the reason for the continuous growth of attention being paid to this drug. Sulforaphane shows an in vitro antiproliferative activity against melanoma and other skin cancer diseases. Unfortunately, this natural compound cannot be applied in free form on the skin due to its poor percutaneous permeation determined by its physico-chemical characteristics. The aim of this investigation was to evaluate ethosomes^®^ and transfersomes^®^ as ultradeformable vesicular carriers for the percutaneous delivery of sulforaphane to be used for the treatment of skin cancer diseases. The physico-chemical features of the ultradeformable vesicles were evaluated. Namely, ethosomes^®^ and transfersomes^®^ had mean sizes <400 nm and a polydispersity index close to 0. The stability studies demonstrated that the most suitable ultradeformable vesicles to be used as topical carriers of sulforaphane were ethosomes^®^ made up of ethanol 40% (*w/v*) and phospholipon 90G 2% (*w/v*). In particular, in vitro studies of percutaneous permeation through human stratum corneum and epidermis membranes showed an increase of the percutaneous permeation of sulforaphane. The antiproliferative activity of sulforaphane-loaded ethosomes^®^ was tested on SK-MEL 28 and improved anticancer activity was observed in comparison with the free drug.

In memory of Prof. Ugo Bottoni, Associated Professor, MD, PhD, Chairman of Dermatology, University School of Medicine “Magna Græcia” of Catanzaro, who collaborated on this scientific work. He prematurely died in January 2018.

## 1. Introduction

Sulforaphane (1-isothiocyanate-(4R)-(methylsulfinyl)-butane) is a natural dietary isothiocyanate. It is an enzymatic product obtained from the reaction between myrosinase and glucopharanin, a 4-methylsulfinylbutyl glucosinolate contained in cruciferous vegetables, e.g., broccoli, brussel sprouts, and cabbage. The attention given to this natural compound is constantly growing due to its multiple pharmacological activities. It has been demonstrated that sulforaphane is effective in brain and neuronal injury with neuroprotective effects [1,2]; in renal [3,4] and liver damage models [5]; in hyperglycemia and β-cell damage [6]; and in damage to the heart, cardiac cells, and other tissues [7]. Sulforaphane is mostly characterized by its potent anticancer activity. In particular, sulforaphane mediates apoptosis and cell cycle arrest; in fact, protein kinases, important mediators of cell growth and proliferation, are inactivated by sulforaphane in tumor cells. Furthermore, it promotes the reactive oxygen species (ROS) production leading to DNA damage and activation of the apoptotic mechanism in different cancer cell lines. Apoptosis can be induced by death receptor 5, activator protein 1, MAP kinases, or mithocondrial dysfunction, or by suppressing the mechanisms responsible for cellular survival, for example, inhibiting activation of the nuclear factor-kappa B [8,9]. Since sulforaphane has these multiple anticancer mechanisms, it is able to inhibit one or more pathways that contribute to malignant transformation and possesses cancer-preventive properties against different tumor diseases [10,11,12], including melanoma [13].

Currently, the treatment of several skin diseases, such as melanoma or actinic keratosis, is very difficult. In fact, single-agent or combination chemotherapy is not sufficient and the application of different strategies to cure, for example, melanoma, seems to be necessary. The topical application of a drug, using a noninvasive route of permeation, may be a good alternative to systemic therapy; in this way, the effective dose can be reduced, the systemic side effects eliminated, and the patient’s compliance increased. Unfortunately, the main limitation in the use of the skin as a route of drug administration is the poor ability of compounds to penetrate the skin barrier, and the physico-chemical properties and stability of natural drugs. For example, free sulforaphane has been demonstrated to be highly unstable both in protic solvents and in polar aprotic environments, so it completely degrades when it is included in conventional pharmaceutical cream formulation [14]. For this reason, an innovative method for inducing percutaneous delivery is required.

Therefore, penetration enhancers that improve percutaneous diffusion are normally used. These substances, such as ethanol, are able to improve the passage through the skin of all components, causing an increased risk of collateral effects. To avoid these problems, topical drug delivery systems, such as ethosomes^®^ and transfersomes^®^, have been developed [15]. Ethosomes^®^, so called for the presence of ethanol in their composition, are deformable vesicular carriers that were developed by Touitou [16]. Ethosomes^®^ are made up of phospholipids, ethanol, and water, and have a greater ability to pass through the stratum corneum barrier and a greater encapsulation efficiency for both hydrophobic [17] and hydrophilic drugs [18]. The high encapsulation efficiency of lipophilic drugs is due to the particular multilamellar structure of ethosomes^®^ [16]. Ethosomes^®^ exhibit a greater fluidity, malleability, and elasticity than conventional liposomes. These features are due to the presence of ethanol in the ethosomal composition, which permits soft systems that can interact with stratum corneum lipids to be obtained, leading to a temporary disorganization of stratum corneum alchilic chains [19] that promote drug percutaneous penetration across the stratum corneum barrier.

Transfersomes^®^, the so-called ultradeformable liposomes, were described for the first time by Cevc et al. [20]. Similar to ethosomes^®^, the deformability of transfersomes^®^ is due to their particular composition. They are made up of phospholipids and sodium cholate, which acts as an edge activator by decreasing the interfacial tension and hence increasing the deformability of the vesicle bilayers [15]. Thanks to their great deformability, the intact permeation of transfersomes^®^ through the skin owing to an aqueous transmembrane osmotic gradient in non-occlusive conditions has been described [21].

When a drug is encapsulated in nanosystems, such as ethosomes and transfersomes, its interaction with the skin depends on the formulation, and no longer on the drug. These topical systems are able to overcome the limits of percutaneous administration of the drug. In particular, the size of the nanosystems can be maintained under 300 nm in order for them to be carried into the deeper layers of the skin [22]. Moreover, negatively charged nanosystems can easily and effectively interact with the skin [23].

The aim of this work was to investigate ethosomes^®^ and transfersomes^®^ as potential carriers for the percutaneous delivery of sulforaphane, in order to propose an innovative clinical therapeutic treatment for skin cancer diseases. The ethosomes^®^ and transfersomes^®^ were characterized from a physico-chemical and technological point of view (i.e., mean size, size distribution, zeta potential, storage stability, entrapment efficacy, and drug release were evaluated), and ex vivo permeation through the human stratum corneum and epidermis membrane was also investigated.

## 2. Materials and Methods

### 2.1. Materials

Phosphatidylcholine (Phospholipon 90 G^®^ - PL90G) (93.0 ± 3.0% phosphatidylcholine) was obtained from Nottermann Phospholipid GMBH, Köln, Germany, and was used without any purification. Absolute ethanol, sodium cholate hydrate (SC) (minimum purity of 99%), Trypan Bleu dye solution (0.4% *v/v*), a 3-[4,5-dimethylthiazol-2-yl]-3,5-diphenyltetrazolium bromide (MTT) dye test (TLC purity ≥97.5%), sodium dimethyl sulfoxide (DMSO), phosphate buffer (PBS) solution, and DL-Sulforaphane (SFN) were purchased from Sigma-Aldrich (Milan, Italy). Eagle’s Minimum Essential Medium (EMEM) culture, fetal bovine serum (FBS 10×), penicillin (100 UI/mL)-streptomycin (100 μg/mL) solution (1% *v*/*v*), and Trypsin/EDTA (1×) solution were obtained from GIBCO (Invitrogen Corporation, Giuliano Milanese (Mi), Italy). Spectra/Por cellulose membranes (Cut off 10,000), used for drug release tests, were provided by Spectrum Laboratories Inc. SK-MEL-28, human melanoma cells, were provided by the Istituto Zooprofilattico of Modena and Reggio Emilia, Italy. Double distilled pyrogen-free water was used throughout experimental investigations. All other materials and solvents used in this study were of analytical grade.

### 2.2. Preparation of Ethosomes^®^

Ethosomes^®^ were made up of PL90G, ethanol, and water in the percentages reported in Table 1. The ethosome^®^ colloidal suspensions were prepared as previously described [16]. Briefly, PL90G was poured into a hermetically-sealed Pyrex^®^ glass vial and solubilized with a suitable amount of ethanol under mixing at 700 rpm with a magnetic stirrer (Midi MR1 Digital IkamagR; IKA-WERKE GMBH and Co., Staufen, Germany). Double distilled water was slowly added at 25.0 ± 0.1 °C. The obtained ethosomes^®^ were then homogenized at 15,000 rpm for 1 min using an Ultra-Turrax T 25 equipped with an S25 N-8G homogenizing probe (IKA-WERKE) and then left at room temperature for 30 min under continuous stirring (Orbital Shaker KS 130 Control, IKA-WERKE). To achieve sulforaphane-loaded ethosomes^®^, the drug (55 µg/mL) was dissolved in ethanol during the preparation procedure of the various formulations.

### 2.3. Preparation of Transfersomes^®^

Transfersomes^®^ were made up of PL90G, SC, and water (Table 1) and were prepared using the thin layer evaporation method as previously reported [24]. Briefly, PL90G and SC were dissolved in a round-bottomed flask with absolute ethanol. The flask containing lipid solution was connected to a rotary evaporator (Rotavapor^®^ R-210, Büchi-Italia, Milan, Italy) at 60 °C and under a slow nitrogen flux, until all traces of solvent had evaporated and a thin-layer lipid film had formed. An ethanol/water (7:93 *v*/*v*) mixture (final volume equal to 6 mL) was added to the lipid film and a vesicular colloidal suspension was obtained by mixing at 700 rpm for 15 min using an orbital Shaker (KS 130 Control, IKA-WERKE). The carriers were left at 40 °C for 2 h in order to stabilize the obtained suspension. Transfersomes^®^ were then subjected to the extrusion procedure through polycarbonate membranes (400 and 200 nm), as reported in a previous investigation [25]. To achieve sulforaphane-loaded transfersomes^®^, the drug (55 µg/mL) was dissolved in ethanol during the preparation procedure of the various formulations.

### 2.4. Physico-Chemical Characterization of Vesicle Formulations

The mean size, size distribution, and z-potential were evaluated by a Zetasizer Nano ZS (Malvern Instruments Ltd., Worchestershire, United Kingdom), following a 1:50 dilution of the samples. Zetasizer Nano is a dynamic light-scattering spectrophotometer (DLS) and a third-order cumulant fitting correlation function was used for sample analysis. This instrument was equipped with a 4.5 mW laser diode operating at 670 nm, which was the light source; the back-scattered photons were detected at 173°. Before starting the analyses, the medium refractive index (1.330), medium viscosity (1.0 mPa × s), and dielectric constant (80.4) were set. The samples were placed in quartz cuvettes to be analyzed [26].

To evaluate the stability of the prepared vesicular carriers, Turbiscan Lab^®^ Expert, equipped with a Turbiscan Lab Cooler, was used. The photon transmitted (T) and backscattered (BS) through the samples, placed in a cylindrical glass tube, was detected [27]. The kinetic stability of the samples was evaluated from the data, obtained by TurbiSoft software (Formulaction, L’Union, France). Measurements were taken for 1 h at room temperature (24 ± 1 °C).

### 2.5. Entrapment Efficacy of Ethosomes^®^ and Transfersomes^®^

The amount of entrapped sulforaphane was separated from the untrapped aliquot by using ultracentrifugation. The colloidal vesicles were poured into polycarbonate tubes and then centrifuged at 90,000× *g* for 1 h at 4 °C using an Avanti 30 Centrifuge (Beckman, Fullerton, CA, USA) equipped with a fixed angle rotor Beckman mod. F1202. The supernatant and the pellet were divided and separately analyzed using HPLC (A Jasco PU-1580 intelligent HPLC pump, Tokyo, Japan) (see Section 2.9).

To break the pellets, 4 mL of ethanol was used. Possible interference from vesicle components was avoided using empty ethosomes^®^ and transfersomes^®^ as blanks. To quantify the amount of sulforaphane entrapped in vesicular systems, the difference between the drug used during the preparation and the non-encapsulated drug was calculated. The following equation was used to determine the encapsulation efficiency (EE%) of sulforaphane in deformable vesicles:

(1) $$\mathrm{EE}\%\;=\frac{\mathrm{De}}{\mathrm{Da}}\;\times \;100,$$

where De is the amount (mg) of entrapped sulforaphane and Da is the sulforaphane amount (mg) used to prepare ethosomal and transfersomal formulations. The reported results represent the average value of five different formulations ± standard deviation.

### 2.6. Sulforaphane Release Profiles

Dynamic skin permeation systems (Laboratory Glass Apparatus, Inc. 1200 Fourth Street Berkeley, CA 94710, USA) were used to evaluate the vesicles’ ability to release the sulforaphane and to permeate through the skin. They were characterized by a surface area of 0.75 cm^2^ and a nominal receiving volume of 4.75 mL. They were composed of two compartments, i.e., the donor, filled with vesicular colloidal suspensions (200 µL), and the receptor, filled with an ethanol/water mixture (20:80 *v/v*). For the release studies, a synthetic cellulose membrane (molecular cut-off weight of 10,000 Da) was interposed between the two compartments. Throughout investigations, sink conditions were maintained, the fluid receptor was constantly stirred with a small magnetic stirring bar, and the temperature was maintained at 32.0 ± 0.5 °C by means of a circulating water bath [28]. The duration of experiments was 24 h and at specific time intervals, 500 µL of the receptor phase was collected for HPLC (see Section 2.9) determination of the released sulforaphane. The amount of withdrawn receptor solution was replaced with the same amount of fresh solution. Experiments were carried out in triplicate and the results were the average of three different experiments ± standard deviations.

### 2.7. Percutaneous Permeation of Sulforaphane-Loaded Deformable Vesicles

Dynamic skin permeation systems were used to evaluate the in vitro percutaneous permeation of sulforaphane in free form and entrapped in deformable vesicles (*ethosomes^®^ and transfersomes^®^*) through human stratum corneum and viable epidermis (SCE) membranes. The study was conducted in accordance with the Declaration of Helsinki, and the protocol was approved by the Research Ethics Committee of the University “Magna Græcia” of Catanzaro (Italy). SCE membranes were prepared as described by Paolino [29], using fresh abdominal human skin obtained from the plastic reduction surgery of healthy adults (mean age 30 ± 4 years). Briefly, the subcutaneous fat was removed by a scalpel and skin samples were put in distilled water for two minutes at 60 ± 1 °C to obtain SCE membranes, which were peeled off from dermis. The obtained sheets (average thickness ~ 40 µm) [30] were stored at 4 °C until being used. As described for release studies (Section 2.5), the receptor compartment was filled with an ethanol/water solution (20:80 *v*/*v*) and stirred at 600 rpm, while the donor compartment was filled with 200 µL of sample. In this case, the SCE membrane was interposed between the two compartments, after its hydration in isotonic sterile saline solution. At prefixed intervals, for all 24 h of the experiments, aliquots of the receptor phase were withdrawn and immediately analyzed by HPLC (see Section 2.9) to determine the amount of permeated sulforaphane. Additionally, in this case, the withdrawn volume was replaced with the same amount of fresh solution and the temperature was maintained at 32.0 ± 0.5 °C by means of a circulating water bath [28].

The skin samples obtained from skin permeation system studies were homogenized for 5 min in the presence of 1 mL of methanol and sonicated (Sonopolus GH70, Bandelin-Electonic, Berlin, Germany) at 50 cycles/s. A following centrifugation procedure was carried out on the tissue suspension for 10 min at 7000 rpm by Megafuge 1.0 Centrifuge (Heraeus Sepatech, Osterode/Harz, Germany). The obtained supernatant was finally analyzed by HPLC to determine the amount of drug trapped in the skin samples.

Experiments were carried out in triplicate and the results were the average of three different experiments ± standard deviations.

### 2.8. Cell Cultures

Plastic culture dishes (100 mm × 20 mm) were used for the incubation of Melanoma cell line SK-MEL-28 in a Guaire^®^ TS Autoflow Codue Water-Jacketed incubator at 37 °C (5% CO_2_). EMEM, containing penicillin (100 UI/mL), streptomycin (100 μg/mL), amphotericin B (250 μg/mL), and FBS (10% *v*/*v*), was used as a medium for cell line growth and replaced with fresh EMEM every 48 h. Trypsin (2 mL) was used to remove the cells adhered to the plate as soon as 80% confluence occurred. Then, 4 mL of the culture medium was placed into a centrifuge tube and centrifuged (1000 rpm) at room temperature for 10 min with an Eppendorf Centrifuge 5810. Finally, before in vitro experiments, fresh EMEM medium was used to resuspend the pellet.

### 2.9. Evaluation of In Vitro Anticancer Activity

The cells were plated in 96-well culture dishes at a density of 10,000 cells/0.2 mL in triplicate, and were then maintained at 37 °C for 24 h before the start of the experiment. After this incubation time, fresh EMEM medium containing different concentrations of free and entrapped sulforaphane was added to the plates with cells in order to replace the culture medium, followed by reincubation for 24, 48, or 72 h. As a control, we used eight wells for each plate with untreated cells. After treatment of the cells with the different samples, 10 μL of MTT (5 mg/mL dissolved in PBS solution) was placed in each well; after 3 h of incubation, supernatants were removed and (200 μL) dimethyl sulfoxide/ethanol solution (1:1 *v*/*v*) was added to each well to solubilize the colored formazan crystals. The plates were gently shaken at 230 rpm (IKA^®^ KS 130 Control, IKA^®^ WERKE GMBH & Co, Staufen, Germany) for 20 min. The ELISA microplate reader (BIO RAD, xMark™ Microplate Absorbance Spectrophotometer) at λ_abs_ 570 nm and λ_em_ 670 nm was used to study the absorbance values of all the analyzed samples. The percentage of cell viability was calculated according to the following equation:

Cell viability (%) = AbsT/AbsC × 100,(2)

where AbsT is the absorbance of treated cells and AbsC is the absorbance of control (untreated) cells. Cell viability values were the average of three different experiments ± standard deviation.

### 2.10. HPLC Analysis

Samples of sulforaphane derived for entrapment efficiency, release profile, and percutaneous permeation studies were quantified using HPLC (A Jasco PU-1580 intelligent HPLC pump, Tokyo, Japan). Chromatographic conditions for reversed-phase HPLC with UV photodiode array detection were as follows: column, Phenomenex Jupiter C18, 5 µm; column temperature, 25 °C; mobile phase, a 30:70 (*v*/*v*) mixture of acetonitrile:water; flow rate, 0.6 mL/min [31]. The UV detection wavelength was 202 nm.

### 2.11. Statistical Analysis

Statistical analysis of all experiments was performed by one-way ANOVA. A posteriori Bonferroni *t*-test was carried out to check the ANOVA test. A *p* value <0.05 was considered statistically significant. Values are reported as the average ± standard deviation.

## 3. Results and Discussion

### 3.1. Physico-Chemical and Technological Characterization of Ethosomes^®^ and Transfersomes^®^

The design and development of a drug delivery system for an innovative purpose require a careful and in-depth investigation regarding the chemical-physical and technological features. Suitable features, such as the mean size, polydispersity index, and zeta-potential, are the basis of pre-formulation studies for the possible development of a new pharmaceutical preparation. In particular, a small vesicle size and negative superficial charge are generally required for topical drug delivery systems selected for therapeutic treatments of skin disorders [32]. For these reasons, deformable vesicles were submitted to light scattering analysis so as to choose the most suitable formulation to be tested in vitro. As shown in Table 2, ethosomes^®^ (formulations A-I) and transfersomes^®^ (formulation J) displayed a narrow particle size distribution (<300 nm). As previously demonstrated and reported [16,18], the mean size of ethosomes^®^ is influenced by the amount of ingredients, i.e., the mean size of ethosomes^®^ decreases when the ethanol amount increases and soybean phosphatidylcoline amount decreases. In our case, the dimensions of ethosomes^®^ remained in a rather narrow range, probably because the effect of ethanol was counterbalanced by the lecithin effect.

**Figure 1:**
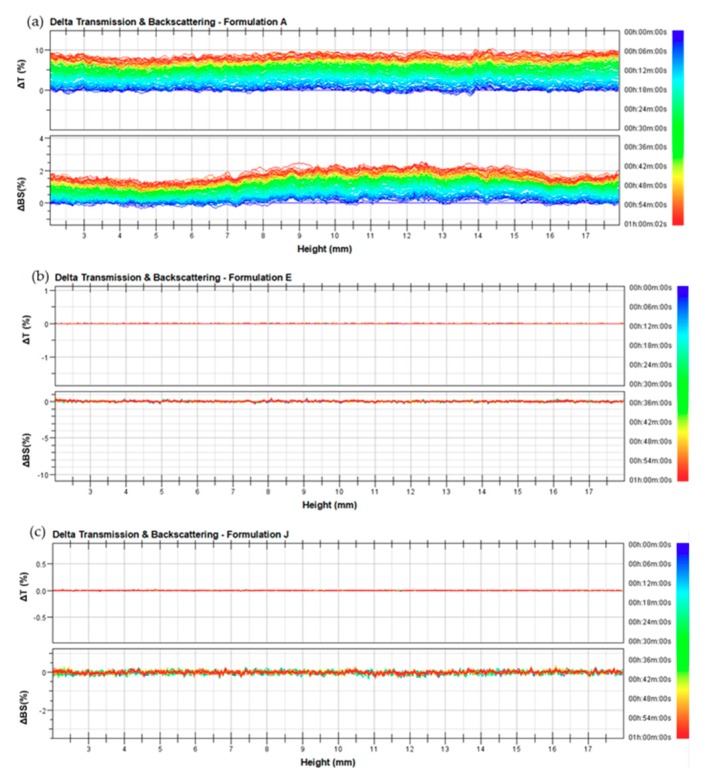
Delta back scattering (ΔBS) and delta transmission (ΔT) profiles of (**a**) formulation A, (**b**) formulation E, and (**c**) formulation J. Panels report representative experiments of five independent experiments. Data are reported as a function of time (0–1 h) and sample height.

All the investigated ethosomal and transfersomal formulations are characterized by negative zeta potential values that are a requirement for topical application and storage stability [16,33]. As shown in Table 2, four formulations (B, D, E, and J) are characterized by suitable zeta potential and polydispersity index values. In fact, a strongly negative zeta potential value and a low polydispersity index should bring no aggregation phenomena, and should thus guarantee a good colloidal stability. Moreover, Turbiscan Lab^®^ Expert analysis [27] was carried out to confirm the DLS results, by measuring the transmission and backscattering profiles as functions of time and sample height. As shown in Figure 1b,c the intensity of back-scattered light of E and J formulations was close to the baseline value during analysis. The Δ transmission and Δ back scattering profiles of B and D formulations are overlapping Figure 1b,c (data not shown). This finding showed that no creaming or sedimentation occurred, thus confirming the stability of the abovementioned nanosystems. The formulations of ethosomes^®^ A, C, F, G, H, and I showed significant variations in their backscattering profiles, thus evidencing a certain destabilization of the colloidal suspension. In Figure 1a, the Δ transmission and Δ back scattering profiles of formulation A are shown as examples of instable profiles.

According to the data obtained through dynamic light scattering and Turbiscan Lab^®^ Expert analysis, the formulations B, D, E, and J were chosen for sulforaphane delivery and subsequent characterization studies.

Drug entrapment within a vesicular carrier is an important parameter to be investigated to really evaluate the topical delivery potentiality of the systems. The entrapment efficiency of sulforaphane within chosen formulations was evaluated. In Table 3, entrapment efficiency values were reported. The chosen formulations were characterized by high drug encapsulation efficiency values, particularly formulations E and J (87.54% and 86.20%, respectively).

The presence of drugs in vesicular systems can influence and modify the physico-chemical characteristics and stability profile of them. In fact, as shown in Table 3, sulforaphane induced a significant increase in the mean size and polydispersity index values of formulations B and D, thus showing a destabilization phenomenon, as evidenced by Turbiscan Lab^®^ Expert analysis.

**Figure 2:**
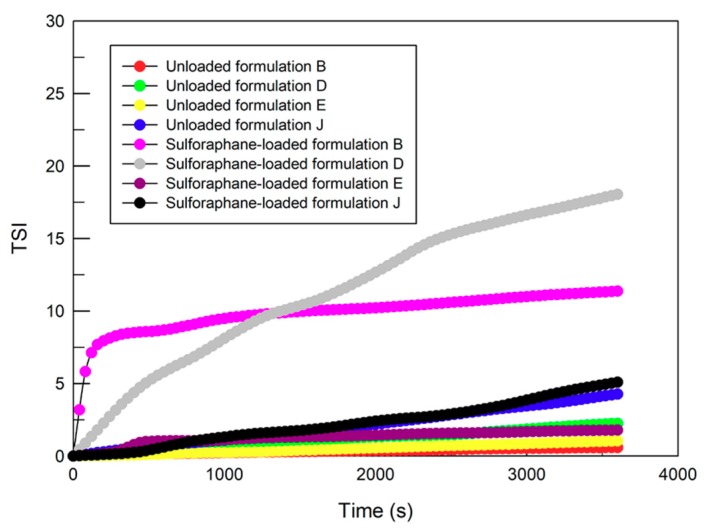
Kinetic stability profiles of unloaded and sulforaphane-loaded ethosomes^®^ and transfersomes^®^ using Turbiscan Lab^®^ Expert. The result was a representative experiment of five independent experiments.

In particular, Figure 2 shows that the profiles of the stability kinetic value of sulforaphane-loaded formulation B and D did not fall within a narrow range of the TSI (Turbiscan Stability Index), thus further supporting the presence of instability phenomena. Instead, E and J samples showed a suitable tolerance with respect to the presence of the drug, by maintaining an unchanged mean size, polydispersity index, and stability profile. Probably, the presence of high ethanol concentrations increases the sulforaphane solubility in the polar phase of the vesicular colloidal formulations. The zeta potential values of the four drug-loaded deformable vesicle formulations were not affected (see Table 3).

### 3.2. Evaluation of Sulforaphane Release and the Percutaneous Permeation Profile

**Figure 3:**
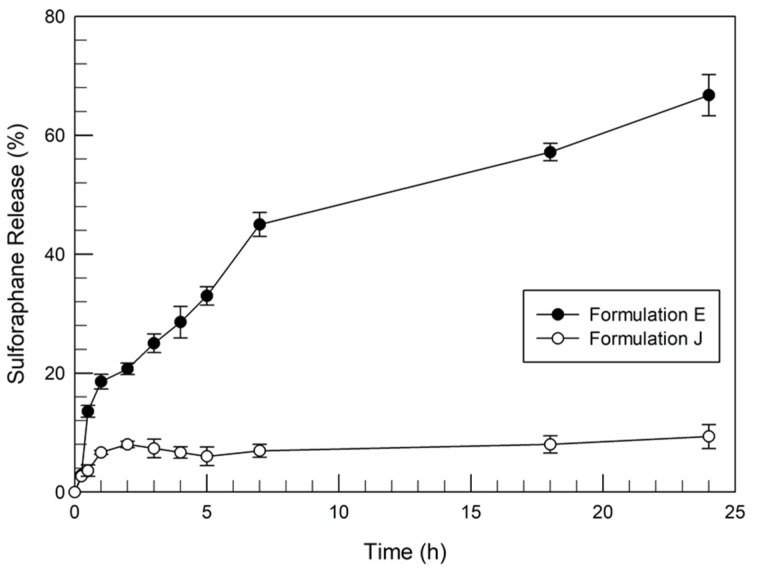
Release profile of sulforaphane entrapped in ethosomes^®^ and transfersomes^®^. Values represent the mean of three different experiments ± standard deviation.

The previously described results allowed the formulations with the best physico-chemical features and the most suitable stability profiles with and without sulforaphane to be selected. The ability of E and J formulations to release the drug was also investigated by using dynamic skin permeation systems, by determining the amount of released sulforaphane as a function of time. As shown in Figure 3, formulation E released ~67% of the entrapped drug during 24 h. The release profile of sulforaphane from formulation E was characterized by a biphasic trend. That is, the amount of sulforaphane entrapped in the outer surface bilayer was quickly released during the first five hours. After this initial rapid release, sulforaphane was gradually and steadily released over time, probably depending on the chemical characteristics of sulforaphane, which allowed a certain affinity with the vesicular structure. On the other hand, the sulforaphane release profile of transfersomes^®^ (Formulation J) was significantly lower (*p* value < 0.005) than the release from formulation E. In detail, this result was less than 10%. This poor release was probably due to the great affinity of sulforaphane to the chemical components (phospholipids and sodium cholate) of the transfersomes^®^, as already demonstrated by Celia et al. [24] for linoleic acid-loaded transfersomes^®^.

**Figure 4:**
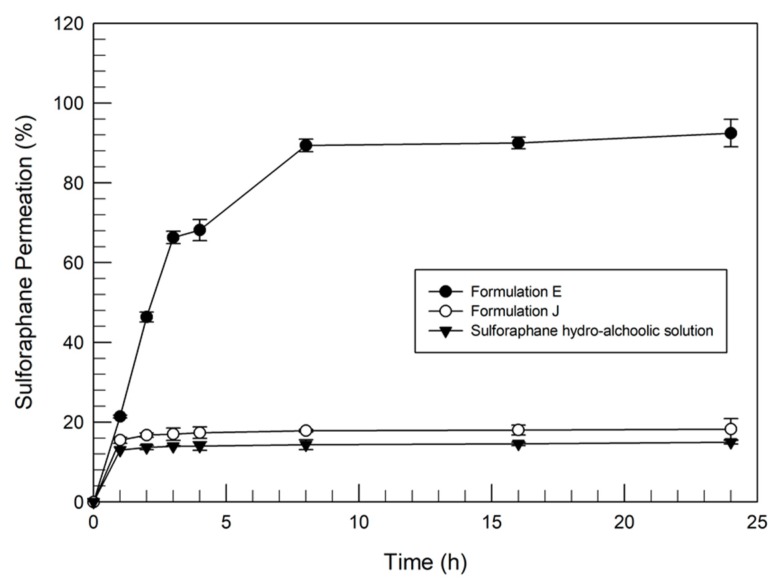
In vitro percutaneous permeation of ethosomes^®^ and transfersomes^®^ containing sulforaphane through SCE membranes, in comparison with a hydroalcoholic drug solution (as the control). Values represent the mean of three different experiments ± standard deviation.

The two formulations (E and J) were further investigated to evaluate their percutaneous permeation through human SCE membranes, in comparison with a water-ethanol solution of sulforaphane. Figure 4 shows that ethosomes^®^ were able to improve the percutaneous permeation of sulforaphane. In particular, the amount of permeated drug was over 90% when delivered by ethosomes^®^. In the case of sulforaphane-loaded transfersomes^®^, the amount of drug detected in the receptor compartment of dynamic skin permeation systems was very low (*p* value <0.001) with respect to sulforaphane-loaded ethosomes^®^, in agreement with the in vitro release studies, and similar to the amount permeated from the hydroalcoholic sulforaphane solution.

The homogenization of skin samples derived from percutaneous permeation studies and the subsequent HPLC analysis confirmed that sulforaphane-loaded transfersomes accumulated in the SCE membrane, with a concentration of sulforaphane equal to 34.14 µg/mL (72 ± 5.8% of the delivered drug).

These findings supported the hypothesis of the interaction and affinity of the drug with the components of the transfersomal formulation. Moreover, the transfersomes^®^ could establish an interaction with the outer structures of the skin, with consequent vesicle accumulation in the corneocytes. This strong interaction could allow systems to be released for more than 24 h. In fact, Celia et al. [24] previously evidenced, following a confocal microscopy study combined with dynamic skin permeation experiments, the interaction and hence the release of transfersomes^®^ from corneocytes after a 48 h incubation. In particular, the confocal microscopy experiments [24] showed that transfersomes^®^ were accumulated in the SCE membrane for a prolonged period of time and then released into the receptor compartment of dynamic skin permeation systems. Based on these findings, our investigation was further developed by choosing, for the following studies, only the ethosome^®^ formulation E.

### 3.3. In Vitro Anticancer Activity of Sulforaphane and Sulforaphane-Loaded Ethosomes^®^

The anticancer properties of sulforaphane-loaded ethosomes^®^ were investigated on human SK-MEL 28 malignant melanoma cells; this cell line was chosen as a model of high proliferative cells. The cytotoxic effects of sulforaphane-loaded ethosomes^®^ were evaluated both as a function of the drug concentration (10, 20, and 50 µM) and the incubation time (24, 48, and 72 h), in comparison with a simple drug solution.

**Figure 5:**
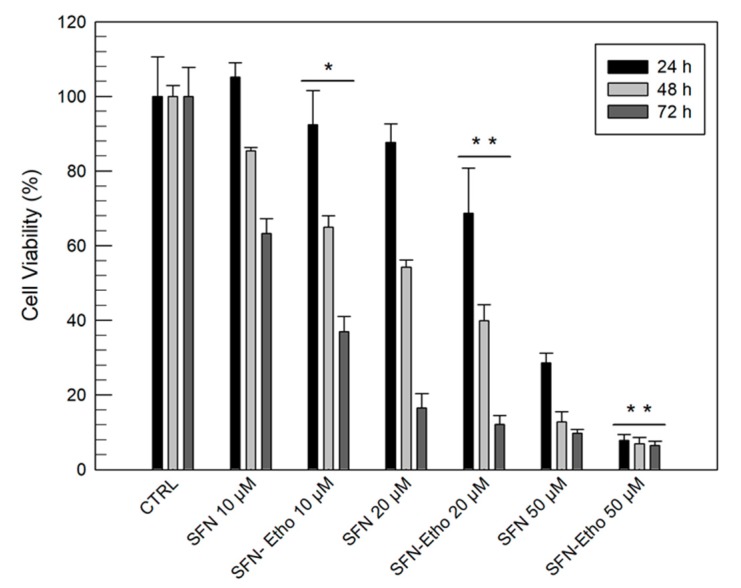
In vitro cytotoxicity of free sulforaphane (SFN) and sulforaphane-loaded Formulation E (SFN-Etho) on SK-MEL 28 melanoma cells as a function of the drug concentration and incubation time. The results were normalized as a function of the blank formulation E cytotoxicity. Results are the mean of three different experiments ± standard deviation. The data obtained for SFN-Etho are statistically significant with respect to the same concentration of free SFN (* *p* < 0.05; ** *p* < 0.001).

The anticancer activity of sulforaphane has been well-described by many studies and the involved mechanisms of action seem to be multiple. Arcidiacono et al. [13], for example, demonstrated that the inhibition of A375 and 501MEL melanoma cells is correlated with reduced AKT phosphorylation induced by sulforaphane. Other studies have shown the proapoptotic effects of sulforaphane on human melanoma cells by p53 and p38 pathways. Moreover, studies have demonstrated that drugs cause G1/S and G2/M cell cycle arrest by altering the levels of cyclin A [13,34,35]. Therefore, the anticancer activity of sulforaphane is due to intracellular mechanisms. Our in vitro results (Figure 5) showed that the ethosome^®^ formulation provided the best anticancer activity on SK-MEL 28 after 24 h and at all tested concentrations compared with the free drug. This trend was also confirmed after 48 and 72 h incubation. The ability of ethosomes^®^ to increase the anticancer activity of the drug is probably due to their fusion with the outer cell membranes, thus allowing cell permeation and drug release directly into the cytoplasm [36].

## 4. Conclusions

The obtained data highlighted the different ability of ethosomes^®^ and transfersomes^®^ to effectively deliver sulforaphane through the skin. The physico-chemical and technological characterization showed that stable sulforaphane-loaded ethosomes^®^ and transfersomes^®^ were obtained, but only one ethosomal preparation showed a suitable ability to contain and release the drug and to successfully permeate the skin. In particular, the different modes of action between ethosomes^®^ and transfersomes^®^ seemed to suggest differences in their efficiency as therapeutic agents for the treatment of melanoma. In fact, ethosomes^®^ elicited an increase of the in vitro percutaneous permeation of sulforaphane compared with transferosomal formulations and drug solutions. This effect is probably due to the presence of ethanol in the composition of ethosomes^®^, which could promote interaction between carriers and lipids of the stratum corneum. These findings are very encouraging and suggest that ethosomes^®^ could be effective carriers for the topical administration of sulforaphane and a real opportunity for the development of an innovative suitable therapeutic strategy for skin cancer disease treatment, such as that for melanoma.

## Figures and Tables

**Table 1:** Lipid composition of ethosomes^®^ and transfersomes^®^**.**

Vesicular Nanocarriers	Formulations		Composition	
		EtOH % (*w*/*v*)	PL90G^®^% (*w*/*v*)	SC % (*w*/*v*)
**Ethosomes^®^**	**A**	30	1	−−
	**B**	30	2	−−
	**C**	30	3	−−
	**D**	40	1	−−
	**E**	40	2	−−
	**F**	40	3	−−
	**G**	45	1	−−
	**H**	45	2	−−
	**I**	45	3	−−
**Transfersomes^®^**	**J**	−−	88	12

**Table 2:** Physico-chemical and technological parameters of blank ethosomes^®^ and transfersomes^®^.

Formulations	Physico-Chemical Parameters		
	Mean Size (nm)	Polydispersity Index	Zeta Potential (mV)
**A**	147 ± 1	0.360 ± 0.052	−12 ± 1
**B**	194 ± 6	0.128 ± 0.002	−28 ± 1
**C**	270 ± 3	0.376 ± 0.067	−20 ± 2
**D**	285 ± 4	0.104 ± 0.023	−25 ± 1
**E**	216 ± 2	0.103 ± 0.003	−26 ± 1
**F**	287 ± 3	0.299 ± 0.034	−19 ± 2
**G**	102 ± 6	0.400 ± 0.087	−21 ± 2
**H**	113 ± 3	0.280 ± 0.008	−20 ± 1
**I**	220 ± 1	0.294 ± 0.056	−17 ± 3
**J**	192 ± 2	0.202 ± 0.011	−30 ± 1

**Table 3:** Physico-chemical and technological parameters of sulforaphane (SFN) ethosomes^®^ and transfersomes^®^ and SFN entrapment efficiency (EE%).

Formulations		Physico-Chemical Parameters		
	Mean Size (nm)	Polydispersity Index	Zeta Potential (mV)	EE(%)
**B**	329 ± 4	0.40 ± 0.02	−28 ± 1	60.4 ± 5.1
**D**	407 ± 4	0.30 ± 0.03	−26 ± 1	67.2 ± 4.1
**E**	227 ± 3	0.01 ± 0.01	−26 ± 1	87.5 ± 2.5
**J**	195 ± 1	0.21 ± 0.02	−30 ± 2	86.2 ± 2.1

## References

[1] Ye Won A., Kyoung A.J., So-Youn W., Jihee Lee K., Young Hae C. (2016). Sulforaphane exerts its anti- nflammatory effect against amyloid-b peptide via STAT-1 dephosphorylation and activation of Nrf2/HO-1 cascade in human THP-1 macrophages. Neurobiol. Aging.

[2] Carrasco-Pozo C., Tan K.N., Borges K. (2015). Sulforaphane is anticonvulsant and improves mitochondrial function. J. Neurochem..

[3] Briones-Herrera A., Avila-Rojas S.H., Aparicio-Trejo O.E., Cristóbal M., León-Contreras J.C., Hernández-Pando R., Pinzón E., Pedraza-Chaverri J., Sánchez-Lozada L.G., Tapia E. (2018). Sulforaphane prevents maleic acid-induced nephropathy by modulating renal hemodynamics, mitochondrial bioenergetics and oxidative stress. Food Chem. Toxicol..

[4] Guerrero-Beltrán C.E., Calderón-Oliver M., Tapia E., Medina-Campos O.N., Sánchez-González D.J., Martínez-Martínez C.M., Ortiz-Vega K.M., Franco M., Pedraza-Chaverri J. (2010). Sulforaphane protects against cisplatin-induced nephrotoxicity. Toxicol. Lett..

[5] Wang W., He Y., Yu G., Li B., Sexton D.W., Wileman T., Roberts A.A., Hamilton C.J., Liu R., Chao Y. (2015). Sulforaphane Protects the Liver against CdSe Quantum Dot-Induced Cytotoxicity. PLoS ONE.

[6] Carrasco-Pozo C., Tan K.N., Gotteland M., Borges K. (2017). Sulforaphane Protects against High Cholesterol-Induced Mitochondrial Bioenergetics Impairments, Inflammation, and Oxidative Stress and Preserves Pancreatic β-Cells Function. Oxid. Med. Cell Longev..

[7] Bai Y., Chen Q., Sun Y.P., Wang X., Lv L., Zhang L.P., Liu J.S., Zhao S., Wang X.L. (2017). Sulforaphane protection against the development of doxorubicin-induced chronic heart failure is associated with Nrf2 Upregulation. Cardiovasc Ther..

[8] Ferreira de Oliveira J.M., Costa M., Pedrosa T., Pinto P., Remedios C., Oliveira H., Pimentel F., Almeida L., Santos C. (2014). Sulforaphane induces oxidative stress and death by p53-independent mechanism: implication of impaired glutathione recycling. PLoS ONE.

[9] Matsui T.A., Murata H., Sakabe T., Sowa Y., Horie N., Nakanishi R., Sakai T., Kubo T. (2007). Sulforaphane induces cell cycle arrest and apoptosis in murine osteosarcoma cells in vitro and inhibits tumor growth in vivo. Oncol. Rep..

[10] Gianfredi V., Nucci D., Vannini S., Villarini M., Moretti M. (2017). In vitro Biological Effects of Sulforaphane (SFN), Epigallocatechin-3-gallate (EGCG), and Curcumin on Breast Cancer Cells: A Systematic Review of the Literature. Nutr. Cancer.

[11] Beaver J.H., Kuintzle L.M., Buchanan R., Wiley A., Glasser M.W., Wong S.T., Johnson C.P., Chang G.S., Löhr C.V., Williams D.E. (2017). Long noncoding RNAs and sulforaphane: a target for chemoprevention and suppression of prostate cancer. J. Nutr. Biochem..

[12] Geng Y., Zhou Y., Wu S., Hu Y., Lin K., Wang Y., Zheng Z., Wu W. (2017). Sulforaphane Induced Apoptosis via Promotion of Mitochondrial Fusion and ERK1/2-Mediated 26S Proteasome Degradation of Novel Pro-survival Bim and Upregulation of Bax in Human Non-Small Cell Lung Cancer Cells. J. Cancer.

[13] Arcidiacono P., Ragonese F., Stabile A., Pistilli A., Kuligina E., Rende M., Bottoni U., Calvieri S., Crisanti A., Spaccapelo R. (2018). Antitumor activity and expression profiles of genes induced by sulforaphane in human melanomacells. Eur. J. Nutr..

[14] Franklin S.J., Dickinson S.E., Karlage K.L., Bowden G.T., Myrdal P.B. (2014). Stability of sulforaphane for topical formulation. Drug Dev. Ind. Pharm..

[15] Cosco D., Celia C., Cilurzo F., Trapasso E., Paolino D. (2008). Colloidal carriers for the enhanced delivery through the skin. Expert Opin. Drug Deliv..

[16] Touitou E., Dayan N., Bergelson L., Godin B., Eliaz M. (2000). Ethosomes – novel vesicular carriers for enhanced delivery: characterization and skin penetration properties. J. Control. Release.

[17] Paolino D., Celia C., Trapasso E., Cilurzo F., Fresta M. (2012). Paclitaxel-loaded ethosomes^®^: Potential treatment of squamous cell carcinoma, a malignant transformation of actinic keratosis. Eur. J. Pharm. Biopharm..

[18] Paolino D., Lucania G., Mardente D., Alhaique F., Fresta M. (2005). Ethosomes for skin delivery of ammonium glycyrrhizinate: in vitro percutaneous permeation through human skin and in vivo anti-inflammatory activity on human volunteers. J. Control. Release.

[19] Paolino D., Cosco D., Cilurzo F., Fresta M. (2007). Innovative drug delivery systems for the administration of natural compounds. Curr. Bioactive Compd..

[20] Cevc G., Gebauer D., Stieber J., Schatzlein A., Blume G. (1998). Ultraflexible vesicles, Transfersomes, have an extremely low pore penetration resistance and transport therapeutic amounts of insulin across the intact mammalian skin. Biochim. Biophys Acta.

[21] Di Francesco M., Primavera R., Fiorito S., Cristiano M.C., Taddeo V., Epifano F., Di Marzio L., Genovese S., Celia C. (2016). Acronychiabaueri Analogue Derivative-Loaded Ultradeformable Vesicles: Physicochemical Characterization and Potential Applications. Planta Med..

[22] Hua S. (2015). Lipid-based nano-delivery systems for skin delivery of drugs and bioactives. Front Pharmacol..

[23] Gillet A., Compère P., Lecomte F., Hubert P., Ducat E., Evrard B., Piel G. (2011). Liposome surface charge influence on skin penetration behavior. Int. J. Pharm..

[24] Celia C., Cilurzo F., Trapasso E., Cosco D., Fresta M., Paolino D. (2012). Ethosomes^®^ and transfersomes^®^ containing linoleic acid: physicochemical and technological features of topical drug delivery carriers for the potential treatment of melasma disorders. Biomed. Microdevices.

[25] De Rose R.F., Cristiano M.C., Celano M., Maggisano V., Vero A., Lombardo G.E., Di Francesco M., Paolino D., Russo D., Cosco D. (2016). PDE5 inhibitors-loaded nanovesicles: Physico-chemical properties and in vitro antiproliferative activity. Nanomaterials.

[26] Cristiano M.C., Cosco D., Celia C., Tudose A., Mare R., Paolino D., Fresta M. (2017). Anticancer activity of all-trans retinoic acid-loaded liposomes on human thyroid carcinoma cells. Colloids Surf. B.

[27] Celia C., Trapasso E., Cosco D., Paolino D., Fresta M. (2009). Turbiscan Lab^®^ Expert analysis of the stability of ethosomes^®^ and ultradeformable liposomes containing a bilayer fluidizing agent. Colloids Surf. B.

[28] Krishnaiah Y.S.R., Pavurala N., Yang Y., Manda P., Katragadda U., Yang J., Shah R., Fang G., Khan M.A. (2017). In vitro drug transfer due to drug retention in human epidermis pretreated with application of marketed estradiol transdermal systems. AAPS PharmSciTech.

[29] Cilurzo F., Cristiano M.C., Di Marzio L., Cosco D., Carafa M., Ventura C.A., Fresta M., Paolino D. (2015). Influence of the supramolecular micro-assembly of multiple emulsions on their biopharmaceutical features and in vivo therapeutic response. Curr. Drug Targets.

[30] Parry G.E., Dunn P., Shah V.P., Pershing L.K. (1992). Acyclovir bioavailability in human skin. J. Investig. Dermatol..

[31] Campas-Baypoli O.N., Sánchez-Machado D.I., Bueno-Solano C., Ramírez-Wong B., López-Cervantes J. (2010). HPLC method validation for measurement of sulforaphane level in broccoli by-products. Biomed Chromatogr..

[32] Ogiso T., Yamaguchi T., Iwaki M., Tanino T., Miyake Y. (2001). Effect of Positively and Negatively Charged Liposomes on Skin Permeation of Drugs. J. Drug Target.

[33] Kohli A.K., Alpar H.O. (2004). Potential use of nanoparticles for transcutaneous vaccine delivery: effect of particle size and charge. Int. J. Pharm..

[34] Fisher M.L., Adhikary G., Grun D., Kaetzel D.M., Eckert R.L. (2016). The Ezh2 polycomb group protein drives an aggressive phenotype in melanoma cancer stem cells and is a target of diet derived sulforaphane. Mol. Carcinog..

[35] Liu K.C., Shih T.Y, Kuo C.L., Ma Y.S., Yang J.L., Wu P.P, Huang Y.P., Lai K.C., Chung J.G. (2016). Sulforaphane induces cell death through G2/M phase arrest and triggers apoptosis in HCT 116 human colon cancer cells. Am. J. Chin. Med..

[36] Godin B., Touitou E. (2004). Mechanism of bacitracin permeation enhancement through the skin and cellular membranes from an ethosomal carrier. J. Control. Release.

